# Landscape of Chimeric RNAs in Non-Cancerous Cells

**DOI:** 10.3390/genes12040466

**Published:** 2021-03-24

**Authors:** Chen Chen, Samuel Haddox, Yue Tang, Fujun Qin, Hui Li

**Affiliations:** 1 School of Basic Medical Sciences, Academy of Medical Sciences, Zhengzhou University, Zhengzhou 450001, China; chenchen320@gs.zzu.edu.cn; 2 Department of Biochemistry and Molecular Genetics, School of Medicine, University of Virginia, Charlottesville, VA 22908, USA; sh7qe@virginia.edu; 3 Department of Pathology, School of Medicine, University of Virginia, Charlottesville, VA 22908, USA; 4 UVA Cancer Center, School of Medicine, University of Virginia, Charlottesville, VA 22908, USA

**Keywords:** chimeric RNA, fusion transcript, gene fusion

## Abstract

Gene fusions and their products (RNA and protein) have been traditionally recognized as unique features of cancer cells and are used as ideal biomarkers and drug targets for multiple cancer types. However, recent studies have demonstrated that chimeric RNAs generated by intergenic alternative splicing can also be found in normal cells and tissues. In this study, we aim to identify chimeric RNAs in different non-neoplastic cell lines and investigate the landscape and expression of these novel candidate chimeric RNAs. To do so, we used HEK-293T, HUVEC, and LO2 cell lines as models, performed paired-end RNA sequencing, and conducted analyses for chimeric RNA profiles. Several filtering criteria were applied, and the landscape of chimeric RNAs was characterized at multiple levels and from various angles. Further, we experimentally validated 17 chimeric RNAs from different classifications. Finally, we examined a number of validated chimeric RNAs in different cancer and non-cancer cells, including blood from healthy donors, and demonstrated their ubiquitous expression pattern.

## 1. Introduction

Chimeric fusion RNAs and their encoded proteins were once thought to be features unique to cancer, some of which have been successfully used as cancer diagnostic markers and therapeutic targets [1,2,3,4]. For instance, *BCR-ABL* in chronic myelogenous leukemia (CML) [5], *TMPRSS2-ETS* [6,7], *SLC45A3-ELK4* [8,9,10,11], and *D2HGDH-GAL3ST2* in prostate cancer [12], *LHX6-NDUFA8* and *SLC2A11-MIF* in cervical cancer [13], *RRM2-C2orf48* in colorectal cancer (CRC) [14], and *ASTN2-PAPPAas* in esophageal cancer [15]. Compared to non-cancer tissues and cells, most of these chimeric RNAs are significantly over-expressed in cancer. Without doubt, they represent effective markers for clinical diagnosis/prognosis, and/or drug targets.

However, recent studies have revealed the existence of chimeric RNAs in non-pathological situations [16,17,18,19,20]. Not only have RNA-Sequencing analyses from normal margins of cancer patients revealed that chimeric RNAs can exist in histologically non-neoplasia tissues [16,17,21,22,23,24,25], an increasing number of paired-end RNA sequencing libraries [26,27,28], such as the Genotype-Tissue Expression (GTEx) dataset [29,30], have also allowed for high throughput discovery of non-cancerous chimeric RNA in normal tissues/cells [20,31]. For instance, *JAZF1-JJAZ1* is observed in endometrial stromal cells and is derived from RNA trans-splicing instead of chromosome rearrangement, which occurs in endometrial stromal sarcoma [32]. *ADCK4-NUMBL*, *C15orf57-CBX3*, *ARL10-HIGD2A*, and other fusions are detected in GTEx samples [31] and knockdown of *ADCK4-NUMBL* and *C15orf57-CBX* decrease cell proliferation and/or cell motility in non-cancerous cells.

In this study, we used three different cell types (HEK-293T, HUVEC, and LO2 cell lines) as models. By interrogating RNA sequencing data via the bioinformatic software tool SOAPfuse, we identified close to four hundred chimeric RNAs. We characterized these chimeras based on various features of the fusions and their parental genes. We then selected 31 candidate chimeric RNAs from different classifications and experimentally validated 17 of these chimeric RNAs as true events in these cell lines. We also explored four of the validated chimeric RNAs in other cells and tissues, including esophageal and prostate cancer and non-cancerous cell lines, as well as healthy blood samples. Additionally, we investigated the location of the transcripts by fractioning cell nucleus and cytoplasm.

## 2. Materials and Methods

### 2.1. Cell Culture

Human embryonic kidney cell line (HEK-293T), non-cancer esophageal cell lines (HEEC, HET-1A, and SHEE) prostate cancer cell lines (LNCaP and 22Rv1), and non-cancer prostate epithelial cell lines (WPMY-1 and RWPE-1) were purchased from Chinese Academy of Sciences (Shanghai, China). Human umbilical vein endothelial cell line, HUVEC, human liver cell line, LO2, and esophageal cancer cell lines, KYSE-140, EC1, Eca-109, were gifts from other laboratories in School of Basic Medical Sciences of Zhengzhou University. HEK-293T, HUVEC, LO2, HEEC, and HET-1A cell lines were maintained in DMEM/HIGH GLUCOSE medium containing 4500 mg/L Glucose and 4.0 mM L-Glutamine (HyClone^TM^, Logan, UT, USA). KYSE-140, EC1, Eca-109, LNCaP, 22Rv1 and WPMY-1 cell lines were maintained in RPMI1640 medium (HyClone^TM^, USA). SHEE was maintained in MEM medium (HyClone^TM^, USA). All mediums mentioned above were supplemented with 10% fetal bovine serum (LONSERA, Ciudad de la Costa Canelones, Uruguay) and 1% penicillin and streptomycin (HyClone^TM^, USA). RWPE-1 cell line was maintained in K-SFM medium (Gibco, USA) without additives. The cells were incubated at 37 °C in a humidified chamber at 5% CO_2_, with medium changes every other day. All cells were digested by 0.25% trypsin with 1 g/L EDTA (HyClone^TM^, USA).

### 2.2. RNA Extraction and qRT-PCR

RNA was extracted from each cell line with BEI-BEI BIOTECH Total RNA Isolation Kit (Zhengzhou, China) and reverse-transcribed by TIANGEN FastKing cDNA Kit (Beijing, China) according to the manufacturer’s instructions. TaKaRa TB Green™ Premix Ex Taq™ (Japan) was used to perform SYBR Green based qRT-PCR experiments. Relative RNA levels were calculated using 2^−(∆∆Ct)^ method. GAPDH was used as internal control.

### 2.3. Real-Time PCR (RT-PCR) and Sanger Sequencing

Candidate chimeric RNAs were validated by RT-PCR. Specific primer pairs for the candidates were designed, with each primer flanking the junction site. All primers used for detection are listed in Supplementary Table S1. RT-PCR experiments were performed with TaKaRa Taq™ Version 2.0 plus dye (Shiga, Japan). Following RT-PCR and gel electrophoresis, Axygen^®^ AxyPrep DNA Gel Extraction Kit (Corning, NY, USA) was used for DNA purification and followed by Sanger sequencing at Sangon Biotech.

### 2.4. Cell Fractionation

HEK-293T cells were digested with 0.25% trypsin, washed once with PBS, and then separated into two fractions using NE-PER nuclear and cytoplasmic extraction reagents (Thermo Fisher, Waltham, MA, USA) following the manufacturer’s instructions. RNA from each fraction was extracted separately with BEI-BEI BIOTECH Total RNA Isolation Kit and followed by qRT-PCR with *MALAT1* and *GAPDH* as controls.

### 2.5. Preparation of Peripheral Blood Buffy Coat

Whole blood samples were obtained from healthy volunteers and stored in EDTA anticoagulant tubes. The blood samples were centrifuged at 1000× *g* for 10 min at 4 °C, and the supernatant plasma above the buffy coat layer was removed and discarded. About 500 µL of buffy coat layer, comprised of white blood cells and platelets, was extracted from erythrocytes and plasma. Total RNA was extracted from the buffy coat with SIMGEN Blood Total RNA Kit (USA) and followed by reverse-transcription and qRT-PCR as described above.

### 2.6. RNA-Seq and Bioinformatics Analysis

RNA-seq was implemented by Novogene (Beijing, China). Common eukaryotic transcriptome library was constructed by the magnetic bead enrichment method. mRNA with Poly(A+) was enriched by magnetic beads with Oligo (dT), then the RNA was broken into 250–300 bp short fragments, which were synthesized into cDNA. The purified double stranded cDNA was repaired, and A tail was added. PCR amplification and purification were performed for the construction of the library. Illumina PE150 sequencing (Pair end 150 bp) was performed. Total reads used for comparative analysis were 59,722,918 in HEK-293T, 59,065,200 in HUVEC, and 71,624,388 in LO2, respectively. The total mapped rate to hg19 was 97.07% in in HEK-293T, 97.24% in HUVEC, and 95.86% in LO2, respectively. Then, we used the bioinformatic software tool, SOAPfuse, to analyze the RNA-seq data and obtain putative chimera lists.

## 3. Results

### 3.1. Discovery of the Chimeric RNAs in HEK-293T, HUVEC, and LO2 Cell Lines

**Figure 1:**
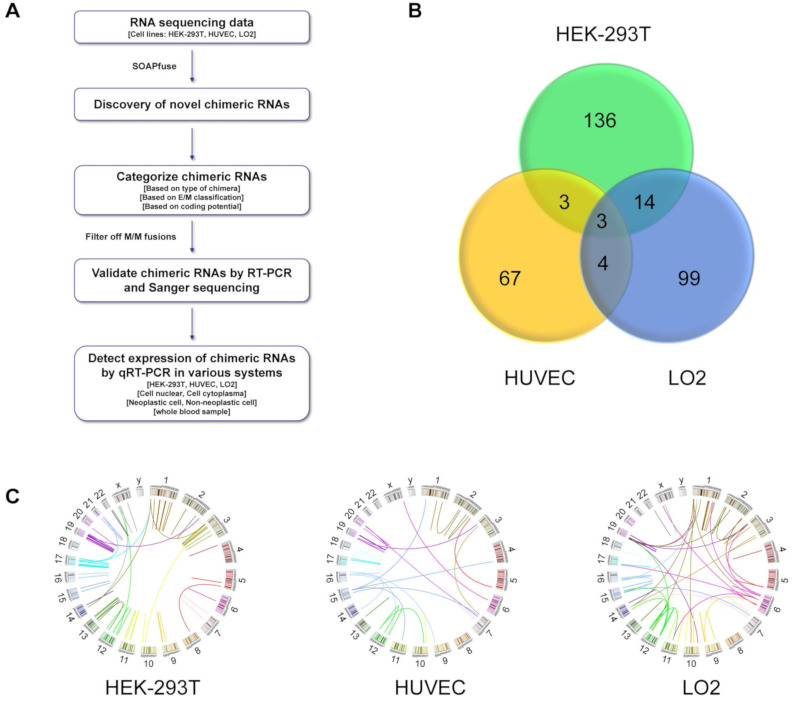
The landscape of chimeric RNAs in HEK-293T, HUVEC, and LO2 cell lines. (**A**) The pipeline for discovering chimeric RNAs. Our own RNA sequencing data was used for analysis. (**B**) Venn diagram summarizing the shared and unique chimeric RNAs that were discovered in HEK-293T, HUVEC, or LO2 cell lines. (**C**) Circos plot depicting chimeric RNAs discovered from three cell lines. The fused genes are illustrated here as a line that connects two parental genes.

To discover chimeric RNAs in non-cancerous cells, we selected three cell lines, representing different cell types. HEK-293T is established from human embryonic kidney, HUVEC is a human umbilical vein endothelial cell line, and LO2 is a human hepatocyte line. RNAs extracted from HEK-293T, HUVEC, and LO2 cells were processed and followed by transcriptome sequencing. To detect and identify novel chimeric RNA events, we used the bioinformatic software tool SOAPfuse [33] to analyze the paired-end RNA-seq data and applied the process outlined in Figure 1A. A total of 156, 77, and 120 unique chimeric fusion transcripts were identified from HEK-293T, HUVEC, and LO2, respectively (Figure 1B,C). The Venn diagram shows the similarities/differences of chimeric RNAs among the cell lines. The overlaps between lines are small, suggesting that most fusion transcripts identified here are cell type specific (Figure 1B). Illustrated by Circos plots, it is apparent that the majority of the fusions are intrachromosomal (Figure 1C). Similar to our previous publication [34], we categorized chimeric RNAs according to junction localization relative to parental genes: both sides being known exon/intron boundaries (E/E), both sides falling into the middle of exons (M/M), and one side being exon/intron boundary and the other not (E/M or M/E). In order to reduce the false discovery rate, we filtered out the M/M-type fusion transcripts because of their lower validation rate [8]. After applying this filter, 118, 32, and 68 unique fusions were left in HEK-293T, HUVEC, and LO2, respectively.

### 3.2. Classifications of Chimeric RNAs

**Figure 2:**
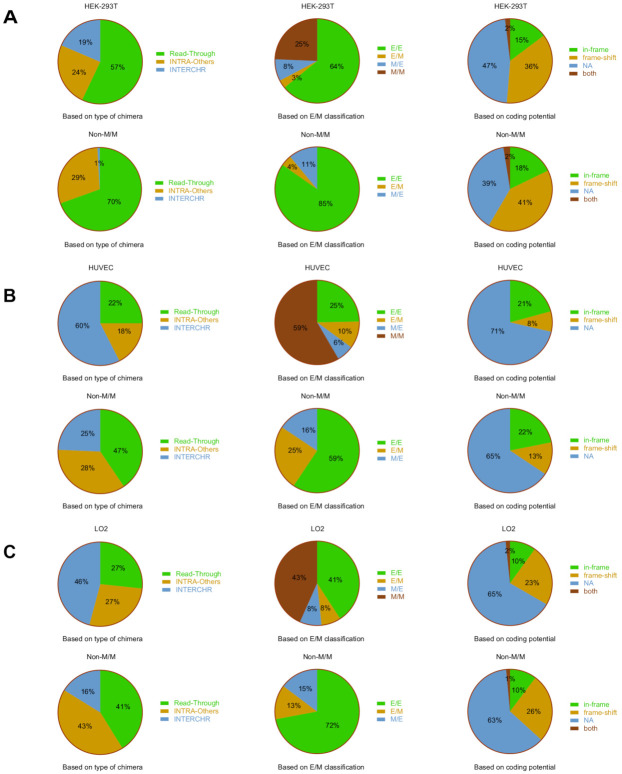
Classifications of the chimeric RNAs in HEK-293T, HUVEC, and LO2 cell lines. Pie charts showing the different distribution of chimeric RNAs in types of chimeras of HEK-293T (**A**), HUVEC (**B**), and LO2 (**C**) cell lines based on parental gene location (left), E/M categories (middle), and fusion protein coding potential (right). The distribution of chimeric RNAs was examined at two stages along our filtering pipeline: all chimeric RNAs and after removal of M/M (Non-M/M). When the criteria of “non-M/M” was applied, more E/E, Read-Through, and in-frame fusions were enriched.

We subsequently examined the landscape of these chimeric RNAs from three angles, and at three different levels, in all three lines (Figure 2A–C). First, we characterized fusions according to chromosomal location of their parental genes: parental genes located on different chromosomes (INTERCHR), neighboring genes on the same strand (Read-Through), and non-neighboring and/or opposite strand genes on the same chromosome (INTRA-Others). For all of the fusions, we found INTERCHR to be the largest group (60% in HUVEC and 46% in LO2) and INTRA-Others to be the least prominent group (18% in HUVEC and 27% in LO2). However, in HEK-293T cells, Read-Through is the most prominent group (57%) and INTERCHR is the least common group (19%). When the M/M-type fusions were filtered out, the INTERCHR group shrunk (1% in HEK-293T, 25% in HUVEC, and 16% in LO2), and Read-Through and INTRA-Others became more abundant (70% and 29% in HEK-293T, 47% and 28% in HUVEC, and 41% and 43% in LO2, respectively).

Secondly, as described above, we categorized chimeric RNAs into M/M, E/M, M/E, and E/E types based on junction location relative to exons of parental genes (Figure 2A–C). Among all the fusions, the most prominent category was E/E fusions: 64% in HEK-293T, while M/M (25%), M/E (8%), and E/M (3%) were much less common in HEK-293T. However, M/M types are more prominent in the other two cell lines (59% in HUVEC and 43% in LO2), while E/E (25% in HUVEC and 41% in LO2), E/M (10% in HUVEC and 8% in LO2), and M/E (6% in HUVEC and 8% in LO2) were much less common. After filtering out the M/M-type, E/E fusions were significantly enriched (85% in HEK-293T, 59% in HUVEC, and 72% in LO2). Interestingly, M/E fusions are always more abundant than E/M fusions in this population of three cell lines.

Lastly, we categorized chimeras according to their reading frames (Figure 2A–C): the known reading frame of the 3′ gene is the same as the 5′ gene (in-frame), the known protein coding sequence of the 3′ gene uses a different reading frame than the 5′ gene (frame-shift), no predicted effect on the reading frame of parental genes (NA) (this category includes chimeric RNAs whose junction sequence fall into an untranslated region, or one or both parental genes is a lncRNA), or a very small population of fusions fell into the “both” category, which could be in-frame or frame-shift, depending on the alternative splicing isoforms of the parental genes. When all the fusions were examined, we found the most common classification to be NA (47% in HEK-293T, 71% in HUVEC, and 65% in LO2), while frame-shift (36% in HEK-293T, 8% in HUVEC, and 23% in LO2) and in-frame (15% in HEK-293T, 21% in HUVEC, and 10% in LO2) fusions were much less common, while the “both” category contained only 2% in HEK-293T and LO2. Removal of M/M-type fusions seemed to enrich in-frame (18% in HEK-293T, 22% in HUVEC, and no change in LO2) and frame-shift (41% in HEK-293T, 13% in HUVEC, and 26% in LO2) classifications, while reducing fusions with NA classification (39% in HEK-293T, 65% in HUVEC, and 63% in LO2).

### 3.3. Validation of the Chimeric RNAs in HEK-293T, HUVEC, and LO2 Cell Lines

**Figure 3:**
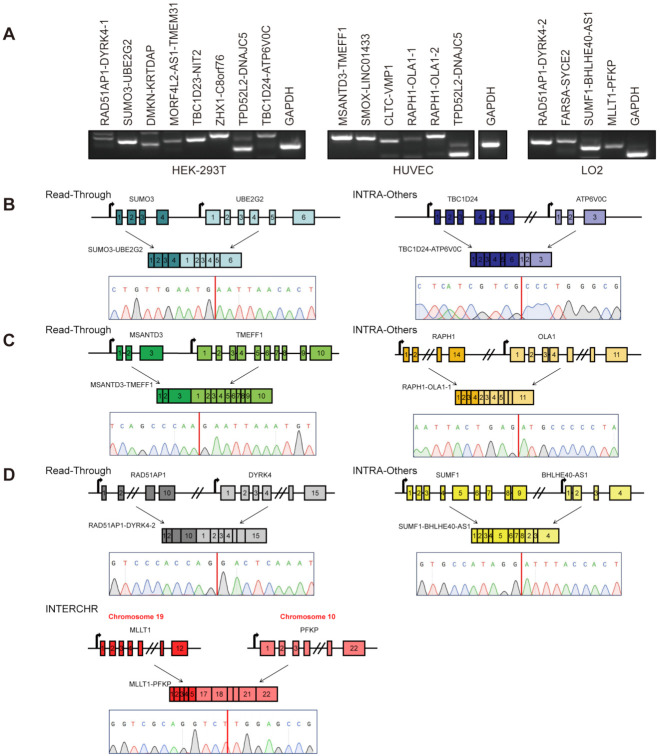
Identification and profiling of Read-Through, INTRA-Others, and INTERCHR chimeric RNA candidates. RT-PCR and Sanger sequencing for candidate chimeric RNAs. (**A**) Gel images of RT-PCR products of eight fusions in HEK-293T, six in HUVEC, and four in LO2 are shown. Structures of one Read-Through, one INTRA-Others and one INTERCHR chimeric RNA and Sanger sequencing validations in HEK-293T (**B**), HUVEC (**C**), and LO2 (**D**). Blocks indicate exons while lines indicate introns and the intergenic region. The fusion junction sites are highlighted as red lines.

Based on the fusion types (Read-Through, INTRA-Others, and INTERCHR), we chose 18, 8, and 5 candidate chimeric RNAs from Read-Through, INTRA-Others, and INTERCHR groups, respectively, for validation (Table 1). To do so, primers annealing to parental genes and flanking the fusion junction site were designed. Sanger sequencing was used after RT-PCR to confirm the sequence on both sides of the junction site in three cell lines (Figure 3A–D and Supplementary Figures S1 and S2). Ten and six candidates were validated from Read-Through and INTRA-Others, respectively (Table 1, and Supplementary Table S2). Of these, two forms for *RAPH1-OLA1* (Figure 3C and Figure S2), *RAD51AP1-DYRK4* (Figure 3D and Figure S1), and *TPD5212-DNAJC5* were validated in both HEK-293T and HUVEC (Figure 3A). However, only one chimeric RNA, *MLLT1-PFKP,* was confirmed from the INTERCHR group (Figure 3D). Similar to our previous research, the validation rate of chimeric RNAs from INTERCHR group is lower than that of chimeric RNAs from Read-Through and INTRA-Others groups.

### 3.4. Expression of the Candidate Chimeric RNAs in HEK-293T, HUVEC, and LO2 Cell Lines

**Figure 4:**
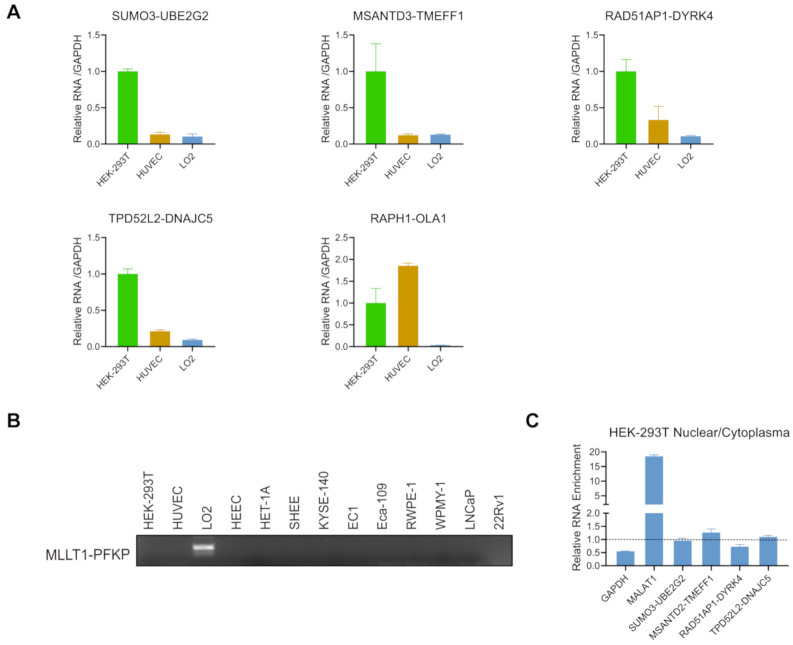
Expression of candidate chimeric RNAs in HEK-293T, HUVEC, and LO2 cell lines. (**A**) qRT-PCR measuring five chimeric RNAs in HEK-293T, HUVEC, and LO2 cells. The levels of various transcripts were normalized to that of *GAPDH*, and further normalized to that in HEK-293T. (**B**) Gel image of RT-PCR products of *MLLT1-PFKP* in 13 cell lines. (**C**) qRT-PCR measuring the expression of four chimeric RNAs in cell nuclei and cytoplasm. HEK-293T cells were fractioned into nuclear and cytoplasmic parts. *GAPDH* and known long non-coding RNA *MALAT1* were used as controls. Ratios of expression in nuclear and cytoplasm parts were plotted.

To quantify the chimeric RNAs discovered, we subsequently measured the expression of six novel chimeric RNAs among these three cell lines by qRT-PCR (Figure 4A). We chose three Read-Through chimeric RNAs (*SUMO3-UBE2G2*, *MSANTD3-TMEFF1*, and *RAD51AP1-DYRK4*), two INTRA-Others (*TPD5112-DNAJC5* and *RAPH1-OLA1*), and one INTERCHR (*MLLT1-PFKP*). All of the chimeric RNAs from the Read-Through and INTRA-Others groups were detected in these three cells, with *SUMO3-UBE2G2* and *MSANTD3-TMEFF1* expressed significantly higher in HEK-293T and *RAPH1-OLA1* expressed significantly higher in HUVEC. The INTERCHR chimeric RNA, *MLLT1-PFKP*, was not found in HEK-293T or HUVEC (Figure 4B).

To gain more insight into the protein coding role of the candidate chimeric RNAs, a fractionation experiment was performed to separate nuclear from cytoplasmic RNAs in HEK-293T. Different from regular protein-coding mRNAs, it has been reported that many lncRNAs reside in the nucleus to regulate transcription [35,36,37]. Since expression of *RAPH1-OLA1* is relatively low, we focused on the examination of the other four fusions. Based on bioinformatics analysis, *MSANTD3-TMEFF1* is in-frame, *RAD51AP1-DYRK4*, and *TPD5112-DNAJC5* are frame-shift, and *SUMO3-UBE2G2* is NA, according to their reading frames which are described above. As seen in Figure 4C, a classic protein-coding gene, *GAPDH*, was enriched in the cytoplasmic fraction, while a classic long non-coding RNA, *MALAT1*, was found predominately in the nuclear fraction. All of these four fusions showed close to one ratio of nuclear vs. cytoplasmic fraction, making them candidates for both categories (Figure 4C). These findings also suggest that assessing protein-coding potential based on the bioinformatic prediction Illumina platform short sequencing may be error-prone. Such prediction would be more reliable with full length sequencing platforms.

### 3.5. Expression of the Candidate Chimeric RNAs in Other Cells/Tissues

**Figure 5:**
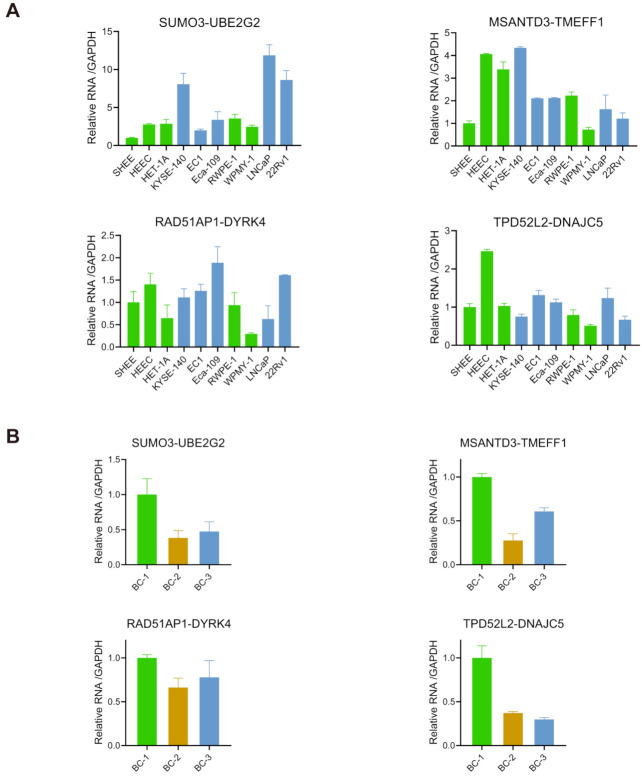
Expression of candidate chimeric RNAs in other cells/tissues. (**A**) qRT-PCR measuring the expression of four chimeric RNAs in various esophageal and prostate cell lines. Blue bars indicate cancer cell lines and green bars indicate non-cancer cell lines. The levels of various transcripts were normalized to that of *GAPDH* and further normalized to SHEE. (**B**) qRT-PCR measuring the expression of four chimeric RNAs in three blood samples (BC-1, BC-2, BC-3). The levels of various transcripts were normalized to that of *GAPDH* and further normalized to BC-1.

To examine the candidate chimeric RNAs in other cells, we performed qRT-PCR for the four fusions in multiple esophageal and prostate cell lines. All four chimeric RNAs were detected in LNCaP and 22Rv1 (prostate cancer cell lines), and RWPE-1 and WPMY-1 (non-cancer prostate epithelial cell lines). They were also detected in KYSE-140, EC1, and Eca-109 (esophageal cancer cell lines), and HEEC, HET-1A, and SHEE (non-cancer esophageal cell lines) (Figure 5A). No significant differences were found between cancer and non-cancer cells.

Lastly, we also examined the expression of these four chimeric RNAs in the buffy coat of three healthy donors. Buffy coat contains various white blood cells and platelets [38]. We extracted RNA from the buffy coat, and conducted qRT-PCR. *SUMO3-UBE2G2*, *MSANTD3-TMEFF1*, *RAD51AP1-DYRK4*, and *TPD5112-DNAJC5* were found in all samples (Figure 5B), supporting that the expression of these chimeric RNAs are more ubiquitous.

## 4. Discussion and Conclusions

Chimeric RNAs caused by chromosomal rearrangements are well known cancer-causing genetic events and are actively used in clinical cancer diagnoses. Some fusion products, such as *BCR-ABL* and *EML-ALK4*, have also shown to be effective targets of directed therapy [39,40]. However, there has been increasing evidence of chimeric RNAs in non-cancer tissues and cells [18,20,31,41]. Recent work on RNA trans-splicing [18,32] and cis-splicing between adjacent genes [8,9,42] have defined a new exemplification for alternative splicing mechanisms, which can also generate chimeric RNAs.

The wide application of RNA-Sequencing has resulted an explosion of newly identified chimeric RNAs. However, it is important to realize that not all chimeric RNAs detected in cancer cells or tissues are cancer specific. Here, we examined the chimeric RNA profile in three different types of non-cancer cells, and uncovered hundreds of chimeric RNAs. Our findings are consistent with the recent realization that chimeric RNAs are not exclusive to cancer cells, and sound alarms for the assumption that all chimeric RNAs identified in cancer are potentially cancer specific biomarkers. This is important as more and more chimeric RNAs are being deposited into Mitelman Database of Chromosome Aberrations and Gene Fusions in Cancer.

In this study, we selected SOAPfuse software to perform paired-end RNA sequencing data analysis because of its high validation rate [8,43]. From the software analysis outcomes, we found small overlaps between three cell lines, supporting that the majority of fusions tend to exist in unique cell types. We performed analyses on the landscape of these chimeric RNAs on three levels and from three angles. After filtering out M/M fusions, the frequency of category INTERCHR shrank significantly. Our previous study also showed that this category of chimeric RNAs tends to have a lower validation rate. Therefore, we decided to omit them from experimental verifications. We randomly selected 31 chimeric candidates from different fusion types (Read-Through, INTRA-Others, and INTERCHR) and confirmed 17 of them. Indeed, we had higher validation for Read-Through and INTRA-Others, with only one INTERCHR chimeric RNA, *MLLT1-PFKP*, verified in LO2. Given the lower validation rate of INTERCHR, it is possible that a subset of them are false positives. Based on bioinformatics analysis, we chose six fusions with significant differences in three cell lines. We noticed that the expression detected by qRT-PCR is not always consistent with the bioinformatic analysis from RNA-Seq data, highlighting the different sensitivity of PCR and sequencing, and the limitation of bioinformatic tools.

In conclusion, instead of being unique features of cancer, chimeric RNAs are widely spread in non-cancer cells. Their ubiquitous expression in many different cells/tissues support them as a set of functional entities involved in fundamental cellular mechanisms common to many cells. Furthermore, tissue specific chimeric RNAs may carry unique functions relevant to the physiological role of the tissue. We believe that chimeric RNAs provide an additional way for the functional genome to expand without an increase in the number of genes. Further study on their function is needed to support this idea.

## Figures and Tables

**Table 1:** Identification of the fusions. Summary of 31 candidate chimeric RNAs. There are two forms for chimeric RNA *RAPH1-OLA1* and *RAD51AP1-DYRK4*. Sanger sequencing confirmed 17 chimeric RNAs noted with *.

Fusion Genes	Sanger Sequencing	Neighboring Genes	Interchromosomal
ARL16-OXLD1	N	Y	N
RAD51AP1-DYRK4-1 *	Y	Y	N
D2HGDH	N	Y	N
SUMO3-UBE2G2 *	Y	Y	N
DMKN-KRTDAP *	Y	Y	N
DPM2-PIP5KL1	N	Y	N
MORF4L2-AS1-TMEM31 *	Y	Y	N
APEH-RNF123	N	Y	N
MFGE8-HAPLN3	N	Y	N
TBC1D23-NIT2 *	Y	Y	N
ZHX1-C8orf76 *	Y	Y	Y
TPD52L2-DNAJC5 *	Y	N	N
TBC1D24-ATP6VOC *	Y	Y	N
MSANTD3-TMEFF1 *	Y	Y	Y
SMOX-LINC012433 *	Y	Y	N
CLTC-VMP1 *	Y	N	N
RAPH1-OLA1-1 *	Y	N	N
RAPH1-OLA1-2 *	Y	N	N
RAD51AP1-DYRK4-2 *	Y	Y	Y
TLCD-RWDD3	N	Y	N
FARSA-SUCE2 *	Y	Y	N
VAMP1-CD27-AS1	N	Y	N
ZNF674-AS1-CHST7	N	Y	N
SUMF1-BHLHE40-AS1 *	Y	N	N
TPCN2-SMIM38	N	N	N
UBXN2A-MFSD2B	N	N	N
MLLT1-PFKP *	Y	N	Y
ASB16-AS1-PHF13	N	N	Y
POLDIP2-TNRC18	N	N	Y
CCDC32-CBX3	N	N	Y
BMERB1-CHP1	N	N	Y

## Data Availability

Data is contained within the article and Supplementary Material.

## Supplementary Materials

The following are available online at https://www.mdpi.com/2073-4425/12/4/466/s1, Table S1. All primers used for RT-PCR. Table S2. Read counts for the validated chimeric RNAs. Figure S1. Identification of chimeric RNA candidates. Figure S2. Identification of chimeric RNA candidates.
