# Supplementary material for: Landscape of Chimeric RNAs in Non-Cancerous Cells
Source: Genes (Basel). 2021 Mar 24;12(4):466. doi: 10.3390/genes12040466 (PMC8064075; doi:10.3390/genes12040466)
Supplement: Supplementary file 1 [file genes-12-00466-s001.pdf]

Table S1. All primers used for RT-PCR. Primers annealing to parental genes of candidate chimeric RNA and flanking the fusion junction site were designed.

TableS1. All primers used for RT-PCR.

Fusion genes	Forward Primer	Reverse primer
RAD51AP1-DYRK4-1	TGAACCAGACTTTGCACCTG	TTCCTTCGCAGAGGTGAACT
SUMO3-UBE2G2	GGCAGCCAATCAATGAAACT	ATTCCTTCCGGAGGATTCAG
DMKN-KRTDAP	GCAGTGGGTGAAGTTTTGGT	CATCCCAGTTGAGGAAAGGA
MORF4L2-AS1-TMEM31	GGCATTAAGGCCAGTGTGTT	TTGTTGGGCTTGAGTTGTTG
TBC1D23-NIT2	AGTCTCGACAAGCGCTGAAT	CTCCCGGATGAAGCTACAAG
ZHX1-C8orf76	GAACCTCCACGACATGTGAA	ACCTTCCTGGACATCCCTTT
TPD52L2-DNAJC5	GAAGACTTCAGCTGCCCTGT	GTTCTTGTCCAACCCAAGGA
TBC1D24-ATP6V0C	CTCCAAGACCGAGTCCATGT	TGTCGTCATTCAGGGAGTTG
MSANTD3-TMEFF1	AAAGTGAAACGGAGCGTCAG	GGCATGCACATTTCAAACC
SMOX-LINC01433	GAGATGCTGCGTCAGTTCAC	ATGAAGGCTGGTGAGCTTGT
CLTC-VMP1	CCAAAGAAGGCAGTGGATGT	GCACCAAAGAAGGTCCAAAA
RAPH1-OLA1-1	TGAGATGTTCCAGCAAGCAG	TGCAGAACGGGAAGTTTTCT
RAPH1-OLA1-2	CCAGCATGGACTCTTTGGAT	TGCAGAACGGGAAGTTTTCT
RAD51AP1-DYRK4-2	AGCTGCCGTCAAATCAGAAT	CTTCCTTCGCAGAGGTGAAC
FARSA-SYCE2	TGAAGAAGTGGGTGGAGGTC	CCGGCTCTTGTTGATGTTTT
SUMF1-BHLHE40-AS1	CCTTCCCTCCCAATGGTTAT	CACAGCCAAACTGCTGAAAA
MLLT1-PFKP	TGACCCCAAGAAGACCAAAC	GGACTGCAGATCCCTGATGTCGA
CTNNBIP1-CLSTN1	ATTCAGCAGAAGGTCCGAGT	TGTGACTATGCCGTGGTAGG
CTNNBIP1	CTCATGCTGCGGAAGATGGGAT	CTGGAAAACGCCATCACCACGT

Table 2. Read counts for the validated chimeric RNAs.

	HEK293T			HUVEC			LO2		
chimeric_RNA	span_reads	junction_reads	total_reads	span_reads	junction_reads	total_reads	span_reads	junction_reads	total_reads
CLTC-VMP1				3	1	4			
DMKN-KRTDAP	13	12	15						
FARSA-SYCE2	2	2	4				2	2	4
MLLT1-PFKP							4	1	5
MORF4L2-AS1-TMEM31	3	3	6						
MSANTD3-TMEFF1				1	2	3			
RAD51AP1-DYRK4-2	4	2	6				1	1	2
RAPH1-OLA1-1				9	2	11			
RAPH1-OLA1-2				7	20	27			
SMOX-LINC01433				2	4	6			
SUMF1-BHLHE40-AS1							3	4	7
SUMO3-UBE2G2	4	5	9						
TBC1D23-NIT2	1	5	6						
TBC1D24-ATP6V0C	4	2	6						
TPD52L2-DNAJC5	3	8	11	2	3	5	5	0	5
ZHX1-C8orf76	3	2	5						

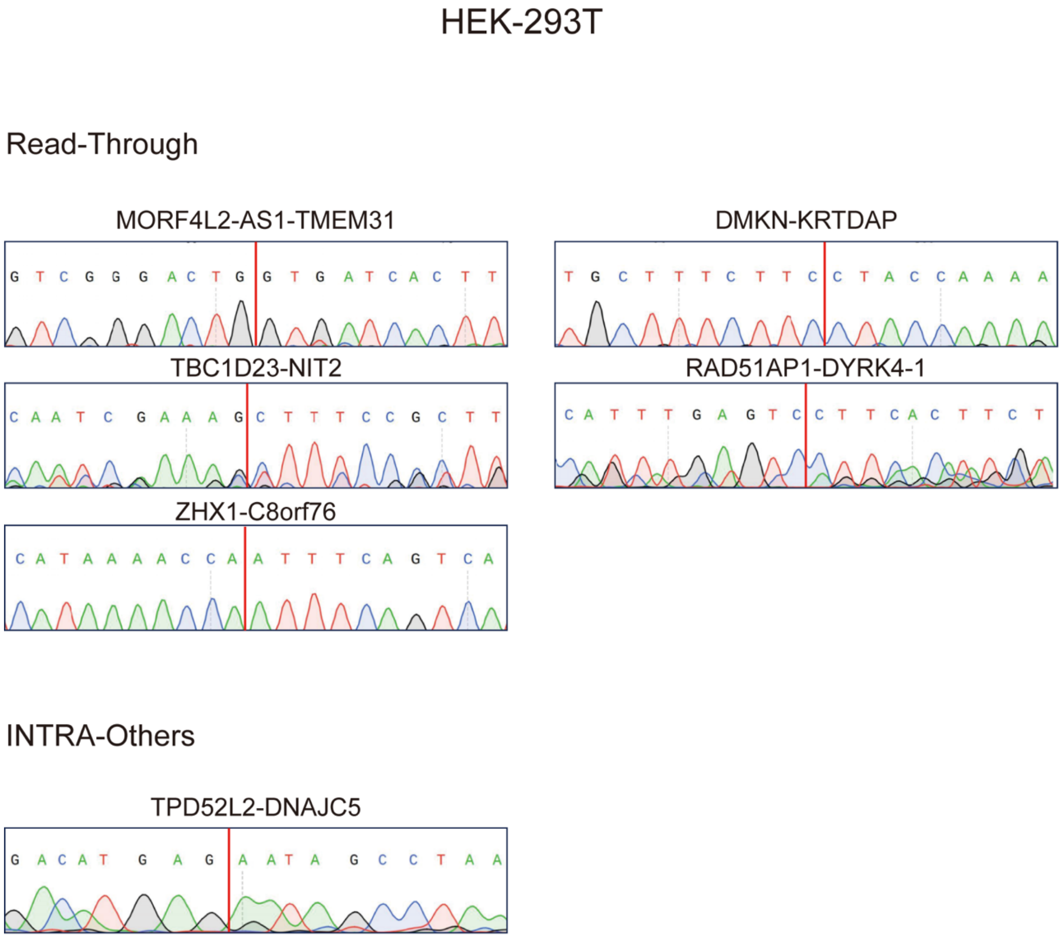

Figure S1

Supplementary Figure S1. Identification of chimeric RNA candidates. Sanger sequencing validation of five Read-Through chimeric RNAs and one INTRA-Others chimeric RNA in HEK-293T.

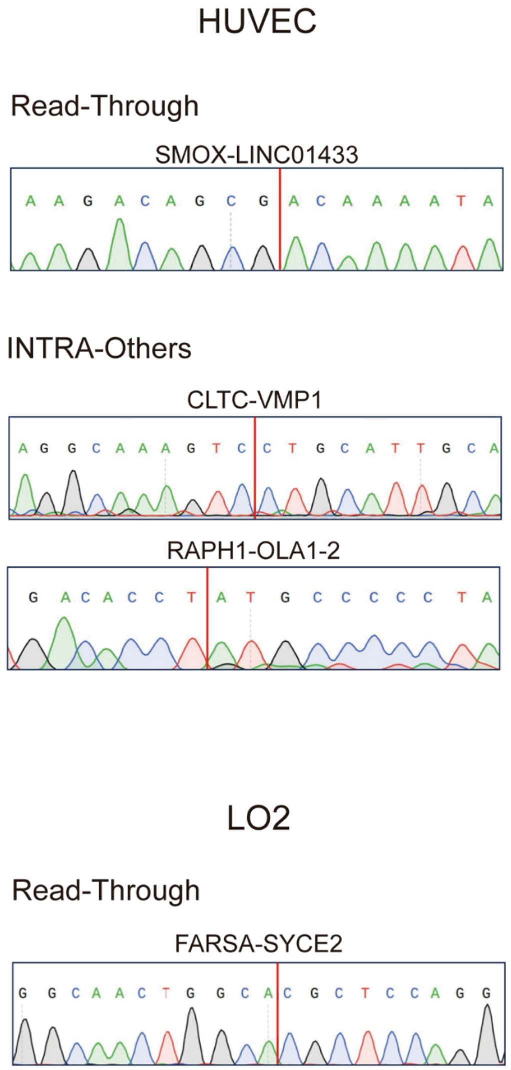

Figure S2

Supplementary Figure S2. Identification of chimeric RNA candidates. Sanger sequencing validation of one Read-Through chimeric RNA and two INTRA-Others chimeric RNAs in HUVEC, and one Read-Through chimeric RNA in LO2.
